# Clinical Remarks upon Surgical Cases in the Buffalo General Hospital—Hydrocele—Operation for Occlusion of Varicose Veins—Tumor from Carotid Region—Tumor from Axillary Space

**Published:** 1869-05

**Authors:** J. F. Miner


BUFFALO

### Medical and Surgical Journal.

VOL. VIII. | MAY, 1869. | No. 10.

### Original Communications.

ART. I.—*Clinical Remarks upon Surgical Cases in the Buffalo General Hospital — Hydrocele — Operation for Occlusion of Varicose Veins—Tumor from Carotid Region—Tumor from Axillary Space.* BY J. F. MINER, M. D.

Gentlemen:—I have opportunity to show this morning a case of hydrocele, which has been sent to me from a neighboring town with the idea that it is diseased testicle, which possibly might better be removed. You will at once see the importance of correct diagnosis in such case, and perhaps appreciate the embarrassment of tapping for hydrocele, a diseased testicle, or worse still, a hernial protrusion of intestine. This case is obscure and not well marked in its characters, and if mistake is ever excusable in case of hydrocele, it might be here, but I am quite sure of my diagnosis. The accumulation is but small, the feeling of fluctuation is indistinct, and it appears unusually hard for hydrocele, but it is not hernia, and the testicle is not enlarged; these conditions being certain, I have no hesitation in introducing trocar and canula for the purpose of demonstrating the accuracy of my diagnosis, and at the same time relieving the ill effects of the disease. Hydrocele signifies a collection of serum in the *tunica vaginalis.* It generally appears in a pear-shaped swelling, smooth on its surface, fluctuating if pressed, free from pain and tenderness, causing only a little uneasiness, mainly from its weight. Diagnosis is very important, and is generally not very difficult. When the *tunica-vaginalis* is filled with clear serum, light held upon the opposite side will show through the tumor with a reddish hue; if, as sometimes occurs, the serum is colored with a small admixture of blood, the tumor is perfectly opaque, and the light test is not available—is liable to mislead; in all instances where the contents of the sac are transparent this test is decisive. It is to be distinguished from hernia, by its forming from below upwards, by its giving no impulse in coughing, by the inguinal canal being unoccupied, and by its history and general appearance. Solid enlargements of the testicle may generally be distinguished from hydrocele by their weight, solidity and tenderness on pressure, and by the absence of fluctuation and transparency; but it must be constantly borne in mind that malignant disease of testicle may have fluid portions, giving rise to fluctuations and possibly to a degree of transparency. This case will not be lost to you if it serve only to impress you with the necessity of correct diagnosis. The treatment is simple and safe. We draw off the serum and inject through the canula tinc. iodine. This is done, as you are all aware, with the view of producing an adhesive inflammation in the membranes of the tunic, causing them to become aglutinated, and thus the sac closed. I think iodine tinc. is as safe and efficient as anything yet proposed, though a great many substances have been recommended. I am in the habit of using it undiluted, as it has much more frequently occurred to me to be obliged to repeat the injection, than to have produced anything of undue inflammatory action. I have never seen any ill effects from its use, and I have never failed after fair trial to obtain the desired effect. There are a variety of forms in which this disease appears, and a great many plans of treatment which surgeons have recommended. I have not now time to mention them, and have simply shown you the operation which adequate experience convinces me is appropriate, safe and successful; there are others, however, which would doubtless secure the same result, and if from any cause you should prefer their adoption, I have no doubt you will be successful in obtaining the desired object.

*Varicose Veins.*—In this condition the veins become enlarged and tortuous, generally thickened, rigid, and divided into irregular pouches, the valves incapable of preventing the reflux of blood. This condition is supposed to be caused by anything which retards the venous circulation; the standing posture, loaded bowels, tumors, or other causes which act to obstruct the vessels. It however, often presents in patients where none of these causes are obvious, and where it seems to arise from constitutional conditions for which we have no very appropriate name. This condition of the veins gives rise to pain, weight and distention while in the erect posture, may cause deep ulcers or produce simple excoriation of the skin. The vessels have been known to burst and produce severe and even fatal hemorrhage; occasionally coagulation of blood takes place in the vessel with subsequent inflammation and abscess.

The treatment may be either *palliative* or *radical.* The palliative consists of support to prevent further enlargement and induce contraction in the distended vessel. This may be effected by bandaging or the use of the elastic stocking; various plans are proposed to effect this object. The radical cure consists in obliteration of the diseased vessel, which may also be effected in several ways. The vein has been subcutaneously divided, a portion of the vessel exsected, caustics applied, pressure made over the vessel, and the circulation arrested by pins and twisted suture. Within the past few years another plan, which I like very well, has been proposed, viz.: the injection of solution of persulphate of iron into the vein with the view of coagulating the blood in the vessel, and thus shutting off the circulation. I propose to show how this operation is made, for upon the details of the procedure depends the success and safety of the operation. The solution is to be diluted largely with water—nine parts water, one part solution persulphate of iron; stronger than this is often used, but it is unnecessary and objectionable. If used in strong solution, it will sometimes act upon the parts and give rise to inflammation of the vessel and surrounding tissues with subsequent abscess, or even sloughing. If by mismanagement the fluid is thrown outside the vessel and is of strong solution, a slough is certain. If you observe my directions you will never throw it elsewhere than you desire it to go, and there will be no ill effects whatever from its use. Open the skin and cellular tissue so as to expose the coats of the vein, which will always be known by the dark blue color.

When this has been carefully and fully done, make pressure so as to obstruct all circulation upon both sides of the point where you propose to introduce the syringe, and inject with an ordinary subcutaneous syringe, a few drops of the solution. You may regulate the extent of the coagulum in the vessel by the points of pressure. I generally make it an inch in extent. Your assistant, who places his fingers above and below your point of injection, should continue his fingers upon the vessel for a minute or so, when the coagulation of the blood will be found to be complete, and the obstruction of the circulation satisfactory. I generally dress the parts with flaxseed poultice, with the view of preventing inflammation, and continue this for two or three days until the cure is complete.

*Tumors* of every variety may be found in the neck, occupying some of the most important surgical regions. Enlarged lymphatic glands, serous and sanguinolent cysts, fatty tumors, adenoid or gland-like tumors, fibro-cystic and cancerous tumors, and other forms of foreign growths are observed in all parts of the neck. It is often quite impossible to determine the exact nature of these growths until after removal, and even then difficult. It is not very important so far as practice is concerned to know their minute characters; the principal and important division is into benign and malignant, and it is generally quite easy to determine if they possess only benign properties; this is, however, not always the case. The growth which we propose to remove is situated in what is called, in surgical anatomy, the "*inferior carotid triangle*," and as you observe its removal depends upon its not being strongly attached to the important vessels upon which it rests. It seems to be cystic or fibro-cystic in character. Certainly it has not the hardness, pain, irregularity and firm attachment so characteristic of malignant growths. It is opened down upon with care, and now its further removal is effected mainly by the handle of the scalpel rather than the edge; in other words, it is peeled out from its bed and removed with comparative ease and safety. If it could not be thus separated from the parts beneath, its removal would be attended with the greatest difficulty and danger.

The remaining operation, which we propose this morning, is for removal of a very large growth which occupies the entire axillary region, extending so high as to press upon the vessels and make it uncertain as to the possibility of safe removal. It has the hard, nodulated, painful and immovable characters which so often accompany malignant disease. The young man looks like the victim of a fatal malady—is pale, anemic and anxious, and we all agree that there are grounds to fear that the tumor is encephaloid—an active form of cancerous disease. However, we propose to remove it, and hope for the best; if it is of this nature, nothing will be gained by the procedure. You observe that it is at last safely removed, though the difficulty has been considerable and the hemorrhage troublesome. All its appearances justify the opinion that it is malignant, and yet its removal is justifiable upon the expectation of delaying its fatal termination; it will not prevent it.

As the lecture term now closes, I may not have opportunity to meet you again in the operating amphitheatre, and I take this occasion to thank you for the earnest attention and deep interest you have all manifested in the clinical advantages of the General Hospital. We have been able to present before you numerous cases of the most important forms of disease, and you have seen the operative treatment which we regarded appropriate. The success attending these operations has been complete, all our patients have recovered from the operation, and all, so far as I know, have received the expected relief. We watch our favorable results with satisfaction, but we must never be unmindful that the best advised and most skillfully made surgical operations will often terminate fatally. They are made with full knowledge that a certain number, often a large proportion, will thus terminate, but we are not at liberty to refuse surgical aid even though attended with the greatest danger; we have seen how even the most unpromising cases reward our efforts the most signally. Hoping those of you who are now to engage in surgical practice, can look back to the clinical experiences of the last season, and gain confidence and strength, I earnestly wish you success in your labors.

---

A Lady Apothecary.—A young woman named Doumergue has just passed an examination before the Academy of Montpelier in the section of pharmacy, and been admitted, with one of the first numbers, to follow the lectures on botany, chemistry, toxicology, and elementary physics.—*Lancet.*

